# The temporal pattern and lifestyle associations of respiratory virus infection in a cohort study spanning the first two years of life

**DOI:** 10.1186/s12887-022-03215-3

**Published:** 2022-03-31

**Authors:** Elizabeth Powell, Edward Sumner, Alex G. Shaw, Ronan Calvez, Colin G. Fink, J. Simon Kroll

**Affiliations:** 1 Section of Paediatric Infectious Disease, Faculty of Medicine, Imperial College London, St Mary’s Hospital Campus, London, W2 1PG UK; 2 Micropathology Ltd, Sir William Lyons Road, Coventry, CV4 7EZ UK; 3 Department for Infectious Disease Epidemiology, School of Public Health, Imperial College London, St Mary’s Hospital Campus, London, W2 1PG UK

**Keywords:** Infants, Respiratory, Virus, Symptoms, Infection

## Abstract

**Background:**

Respiratory virus infection is common in early childhood, and children may be symptomatic or symptom-free. Little is known regarding the association between symptomatic/asymptomatic infection and particular clinical factors such as breastfeeding as well as the consequences of such infection.

**Method:**

We followed an unselected cohort of term neonates to two years of age (220 infants at recruitment, 159 who remained in the study to 24 months), taking oral swabs at birth and oropharyngeal swabs at intervals subsequently (at 1.5, 6, 9, 12, 18 and 24 months and in a subset at 3 and 4.5 months) while recording extensive metadata including the presence of respiratory symptoms and breastfeeding status. After 2 years medical notes from the general practitioner were inspected to ascertain whether doctor-diagnosed wheeze had occurred by this timepoint. Multiplex PCR was used to detect a range of respiratory viruses: influenza (A&B), parainfluenza (1–4), bocavirus, human metapneumovirus, rhinovirus, coronavirus (OC43, 229E, NL63, HKU1), adenovirus, respiratory syncytial virus (RSV), and polyomavirus (KI, WU). Logistic regression and generalised estimating equations were used to identify associations between clinical factors and virus detection.

**Results:**

Overall respiratory viral incidence increased with age. Rhinovirus was the virus most frequently detected. The detection of a respiratory virus was positively associated with respiratory symptoms, male sex, season, childcare and living with another child. We did not observe breastfeeding (whether assessed as the number of completed months of breastfeeding or current feed status) to be associated with the detection of a respiratory virus. There was no association between early viral infection and doctor-diagnosed wheeze by age 2 years.

**Conclusion:**

Asymptomatic and symptomatic viral infection is common in the first 2 years of life with rhinovirus infection being the most common. Whilst there was no association between early respiratory viral infection and doctor-diagnosed wheeze, we have not ruled out an association of early viral infections with later asthma, and long-term follow-up of the cohort continues.

**Supplementary Information:**

The online version contains supplementary material available at 10.1186/s12887-022-03215-3.

## Background

Respiratory viral infection, particularly the importance of asymptomatic viral infection has been a subject of much recent interest secondary to the current COVID-19 pandemic. Detection of respiratory viruses in symptomatic and asymptomatic children has been described in several cohort [1–3] and cross-sectional studies (for example [4, 5]). The strength of association between detection of respiratory viral infection and acute respiratory symptoms is not identical for each respiratory virus. In a recent large paediatric study [6], the association was strongest for respiratory syncytial virus (RSV) and human metapneumovirus (HMPV), whereas in contrast the presence of polyomaviruses (WUPyV and KIPyV), coronaviruses (HCoV-229E and HKU-1) and human bocaviruses (HBoV) were not associated with symptoms. Similar findings were observed in another study which conducted weekly nasal swabs and symptom diaries for families rather than just the infants [7].

The percentage of a defined cohort who have asymptomatic infection with respiratory viruses varies with age - 7% of participants in a neonatal study had an asymptomatic respiratory viral infection (1) compared to 22-68% in studies during infancy and early childhood (2, 3, 8). A temporal pattern with age has also been seen with longitudinal nasal and nasopharyngeal samples (3, 6) with increasing viral infection in the first 9 months of life. Apart from age, other factors such as childcare attendance (3), family size (7) and season of sampling are associated with the presence of respiratory viral infection. Early respiratory viral infection has potential long-lasting consequences [9]. Both symptomatic RSV and rhinovirus infection (for example bronchiolitis or viral induced wheeze) has been associated with later asthma [10]. Less in known regarding any consequence of early respiratory viral infection in the community (i.e., not requiring medical intervention whether asymptomatic or mildly symptomatic) and subsequent health impact.

We sought to establish the pattern of respiratory viral infection over the first two years of life in the oropharynx, the association of infection with symptoms, and the sequelae of infection in relation to early life wheeze. We also aimed to look for evidence of the previously observed protective effect of breastfeeding on respiratory infections in infancy within the cohort.

## Methods

### Recruitment and sampling

The study was approved by the London Riverside Research Ethics Committee reference number 12/LO/1362. The recruitment and sampling of participants has been described elsewhere [11]. In brief, prospective parents were approached in antenatal clinics for assent to be involved in the study, and then were re-approached for written consent once their baby was born. The participants were born in 2013 with 2 years sampling completed by the end of 2015, and medical note collation in 2016. An initial birth interview and oral swab were taken. Participants were then visited at home at 6 weeks, 6, 9, 12, 18 and 24 months of age (and in a subset also at 3 and 4.5 months of age), when a double-headed oropharyngeal swab was taken and a researcher-delivered health questionnaire which included details of respiratory symptoms and medication use was completed. Participants were deemed symptomatic at the time of sampling if there were respiratory symptoms (cough, coryza and/or wheeze (with or without fever)) within the week prior to or after sampling. Swabs from participants whom had other symptoms such as diarrhoea or vomiting (without cough, coryza and/or wheeze) were included and were deemed not to have respiratory symptoms. After the 24 months visit, GP notes were requested in order to determine whether the child had doctor-diagnosed wheeze, which was defined as a recorded diagnosis of wheeze, auscultation recorded as wheeze or mention of wheeze with a prescription of a bronchodilator for the purpose of treating wheeze. The result shown are for all the viral swabs taken (for graphs and tables) or for those who completed the visits to 24 months for statistical analysis.

### Respiratory viral PCR

Material was eluted from swabs in 500 µl 0.1% Igepal CA-630 (Sigma), prior to storage at -80 °C before the next step. Nucleic acid extracts were prepared from 200 µl specimen using a Qiagen MDx Bio Robot according to the manufacturer’s instructions. First round PCR was performed using 20 µl of nucleic acid extract. Multiplex RT-PCR for RNA viruses was carried out using 2 × SensiFAST SYBR No-Rox One Step Mix (Meridian Bioscience) (Additional file 1: S1 table). First round PCR for DNA viruses and second round PCR for all targets was performed using MyTaq™ HS DNA polymerase and mix (Meridian Bioscience). Singleplex second round PCR was performed using 1 µl of first round amplicon as template material. Melt curve analysis using a Roche LightCycler 480 was used for the detection of all PCR products with the exception of bocavirus, which was analysed by agarose gel electrophoresis.

Oligonucleotides were derived from those described in Additional file 1: S1 table and ordered from Sigma. Primers were used at 0.1 μM and 0.2 μM final concentrations for first and second round reactions, respectively. Primers for KI and WU polyomavirus were designed using the NCBI Primer Design tool (http://www.ncbi.nlm.nih.gov/tools/primer-blast/) (see Additional file 1: S2 table). The complete genome sequence from the KI polyomavirus Stockholm 60 (gi|124,366,173|gb|EF127906.1|) was used as reference sequence for the design of the polyomavirus KI primers. The target is a region located between the VP1 and the small T antigen of the virus. The target for WU polyomavirus is the VP1 gene; the full genome sequence of strain WU Polyomavirus strain B0 complete genome sequence (gi|146,199,082|gb|EF444549.1|) was used as a reference sequence.

### Statistical analysis

Statistical analysis was undertaken using r within r studio [12]. Data for participants who did not complete up to the 24 months visit is shown in figures and tables, however statistical analysis was completed only on data from those for whom there was follow up to 24 months. Earlier data from those who were either lost to follow up or withdrew was excluded from the analysis. Participants who did not have outcome data at 24 months for doctor-diagnosed wheeze were excluded from the analysis looking at this outcome measure. Each positive respiratory viral detection was treated as a new infection for that infant. Logistic regression and generalised estimating equations were used to assess for associations between viral detection and clinical factors. Logistic regression was conducted using the ‘glm’ function in the stats package within r. Generalised estimating equations were used where there was longitudinal data using the ‘geepack’ package within r [13]. For multivariable analysis, univariate analysis was first conducted, and independent variables taken forward where the *p* value remained significant following correction for multiple testing. The 95% confidence intervals are presented where applicable. To explore any association between breastfeeding and respiratory viral infection, the variable completed months of breastfeeding was used in the analysis. Any association was then further assessed using the variable current feeding (breast, mixed, formula or weaned (where the infant had commenced solid feeds)).

## Results

### Participant characteristics

The characteristics of the study participants are shown in Table 1.

**Table 1:** The characteristics of participants in the cohort by birth and those followed to 24 months.

**Characteristic**		**Birth** **(***n*** = 220)**	**24 months** **(***n*** = 159)**
**Mode of delivery**	C/S^a^ Vaginal	57 (26%) 163 (74%)	46 (29%) 113 (71%)
**Season of birth**	Winter Spring Summer Autumn	29 (13%) 87 (40%) 77 (35%) 27 (12%)	21 (13%) 69 (43%) 52 (33%) 17 (11%)
**Neonatal antibiotics**	No Yes	202 (92%) 18 (8%)	147 (92%) 12 (8%)
**Intrapartum antibiotics (excluding delivery)**	No Yes	195 (89%) 25 (11%)	139 (87%) 20 (13%)
**Ethnicity**	Asian/Asian British Black/Black British Mixed Other White	23 (10%) 21 (10%) 44 (20%) 26 (12%) 106 (48%)	13 (8%) 11 (7%) 35 (22%) 15 (9%) 85 (53%)
**Birth feed**	Breast Mixed Formula	132 (60%) 82 (37%) 6 (3%)	96 (60%) 61 (38%) 2 (1%)
**Smoker at home (birth)**	Yes No Unknown	53 (24%) 166 (75%) 1 (0%)	35 (22%) 124 (78%)
**Furry Pet at home**	Yes No Unknown	28 (13%) 191 (87%) 1 (0%)	20 (13%) 39 (87%)
**Crowding index**^**b**^** (mean)**	Mean Unknown	0.87 2 participants	0.90
**Parental history of doctor-diagnosed atopy**	Yes No Unknown	99 (45%) 109 (50%) 12 (5%)	81 (51%) 74 (47%) 4 (3%)

^a^C/S = Caesarean Section

^b^Crowding index = number of rooms (excluding kitchen and bathroom) divided by the number of people living there

### Respiratory viral infection

**Fig. 1:**
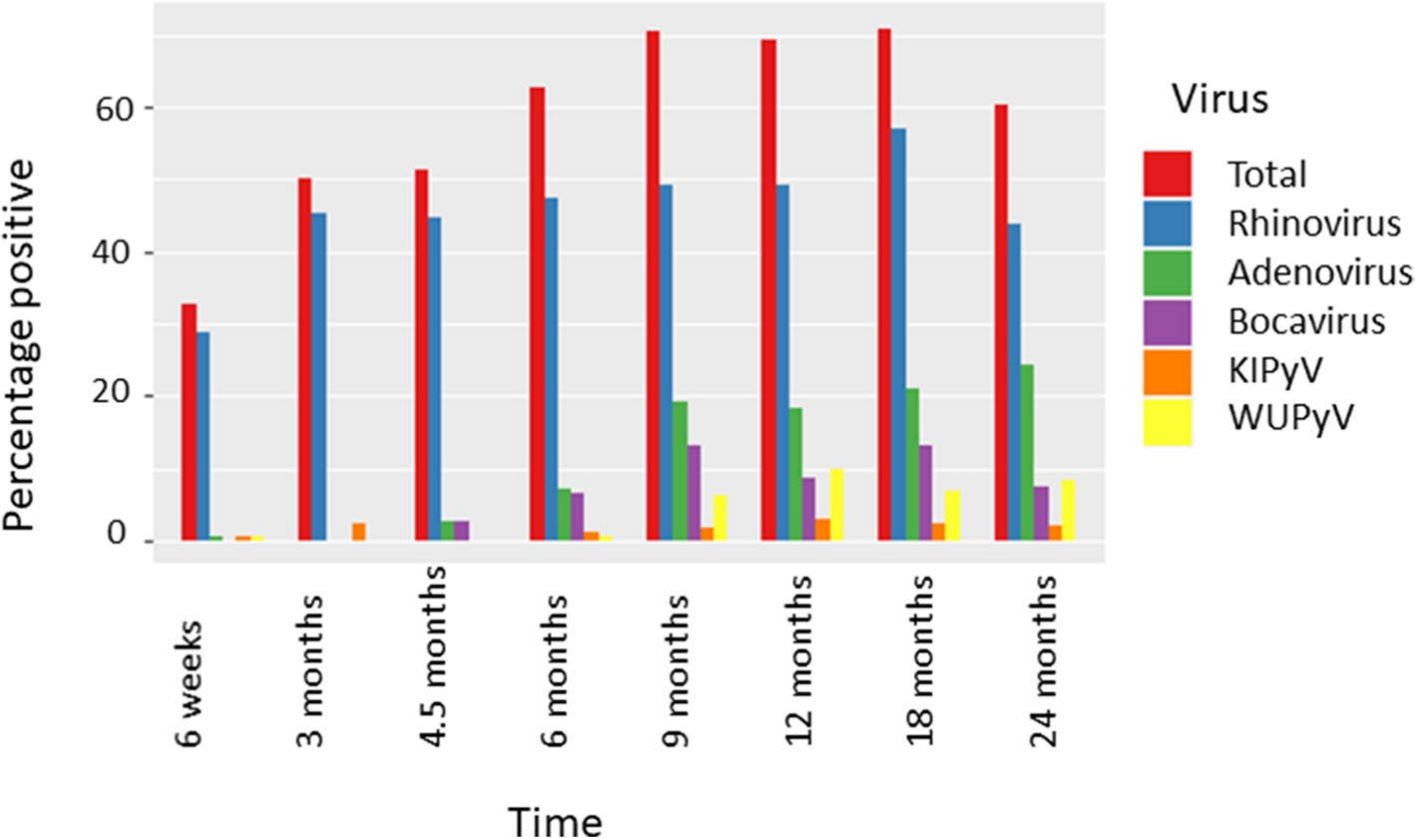
The percentage of swabs positive for the most abundant respiratory viruses by timepoint. Bars indicate percentage of collected samples at each timepoint that tested positive for indicated viruses

**Fig. 2:**
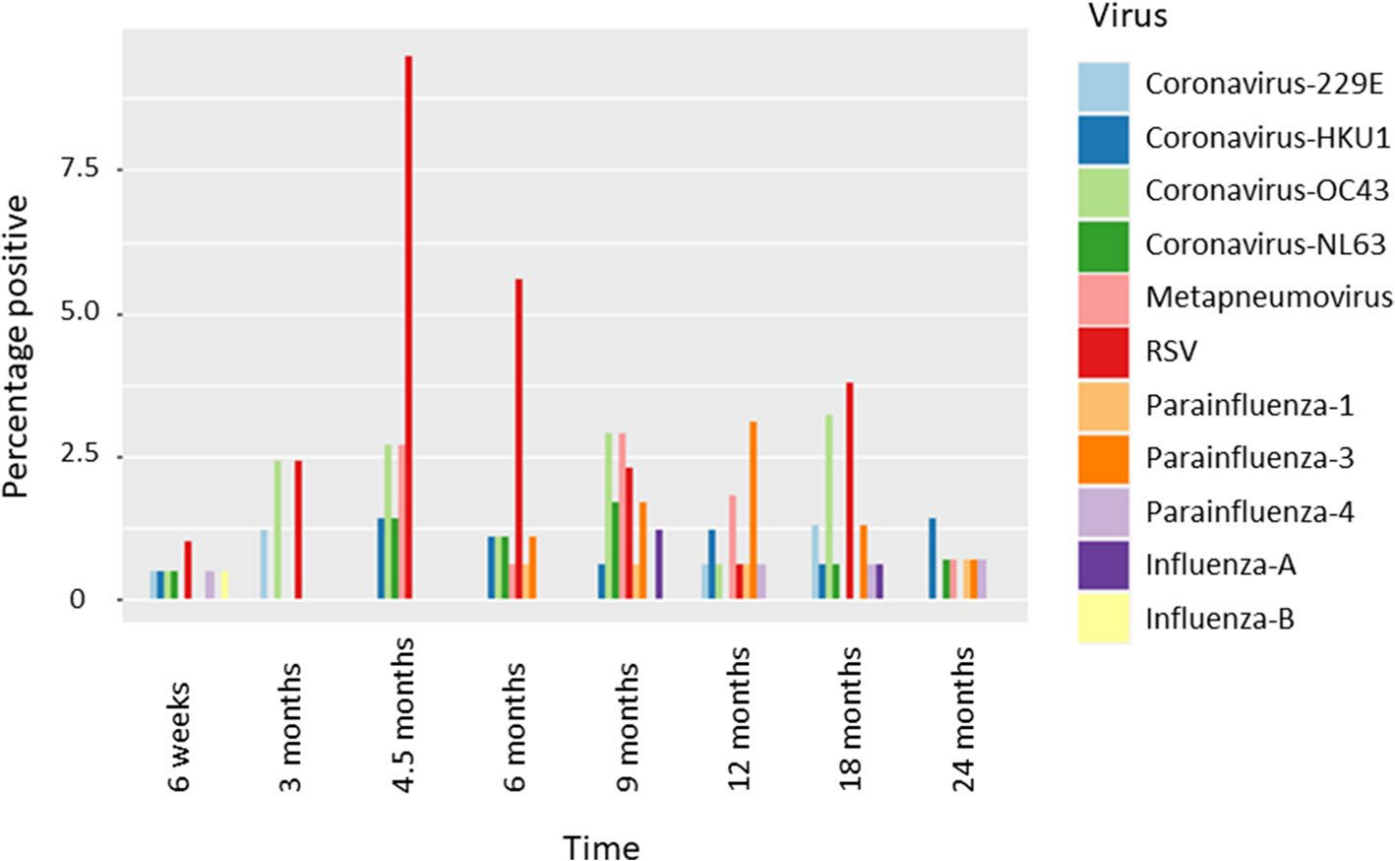
The percentage of swabs positive for the least abundant respiratory viruses by timepoint. Bars indicate percentage of collected samples at each timepoint that tested positive for indicated viruses

Respiratory viral infection was a frequent finding in the cohort participants. Overall, 50.5% of the swabs from birth to 2 years were positive for a respiratory virus. Respiratory viral infection increased with age (*p* < 0.001 association between virus detection and age, GEE model), particularly noticeable up to 9 months (Fig. 1), (Table 2). The temporal pattern was different depending on the respiratory virus – adenovirus and rhinovirus for example increased with age (*p* = 0.013 association of rhinovirus with age, *p *< 0.001 adenovirus with age) whereas other viruses such as human parainfluenza virus (HPIV) and HMPV did not show a trend with age (*p* = 0.071 HPIV, *p* = 0.850 HMPV) or a negative association with age for RSV (*p* = 0.008) (Fig. 2).

**Table 2:** The number of birth oral swabs and infant/child oropharyngeal swabs which tested positive for each respiratory virus

Number of samples tested	**Birth**^**d**^ 215	**6wk** 204	**3mo** 84	**4.5mo** 74	**6mo** 179	**9mo** 173	**12mo** 163	**18mo** 158	**24mo** 144	**Total**
Any^a,b^	11	67(27)	42(17)	38(17)	112 (47)	122(57)	113(50)	112(61)	87(35)	704 (311)
Rhinovirus	6	59(26)	38(16)	33(14)	85(34)	85(43)	80(39)	90(52)	63(29)	539 (253)
Adenovirus	0	1(0)	0(0)	2(1)	13(9)	33(14)	30(10)	33(21)	35(15)	147 (70)
Bocavirus	1	0(0)	0(0)	2(1)	12(4)	23(11)	14(8)	21(11)	11(4)	86 (39)
Coronaviruses^c^	3	4(0)	3(0)	4(3)	6(3)	9(3)	4(2)	9(4)	3(1)	45 (16)
Polyomaviruses^c^	1	2(0)	2(1)	0(0)	3(0)	14(4)	21(7)	10(7)	15(7)	68 (26)
Metapneumovirus	0	0(0)	0(0)	2(2)	1(1)	5(4)	3(1)	0(0)	1(1)	14 (9)
RSV	0	2(1)	2(2)	7(5)	10(6)	4(3)	1(1)	6(5)	0(0)	32 (23)
Parainfluenza^c^	0	1(1)	0(0)	0(0)	3(2)	4(2)	7(6)	3(2)	3(3)	21 (16)
Influenza^c^	0	1(0)	0(0)	0(0)	0(0)	2(1)	0(0)	1(1)	0(0)	4 (2)

^a^For each virus the number positive for each virus and the number symptomatic is shown in the brackets

^b^In the case of a positive test for more than one virus, all are included for each of the virus group they tested positive

^c^For the purposes of the summary table the virus subtypes have been grouped together

^d^Birth samples were taken at a mean of 1 day (range 0 to 9 days)

**Fig. 3:**
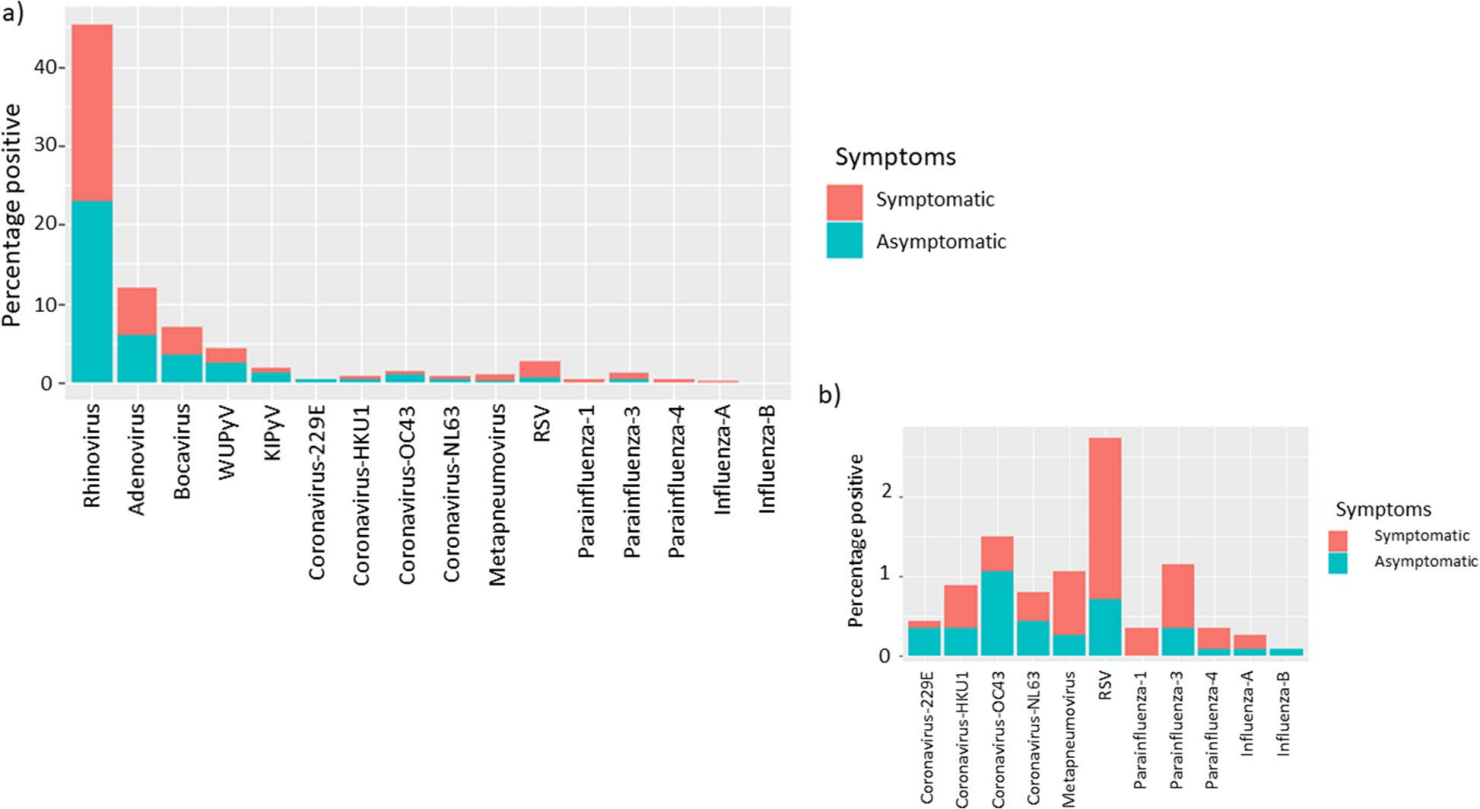
A stacked bar chart to show the percentage of swabs taken from participants which were positive for each virus a) all results b) a ‘zoomed in’ version of the lower abundance viruses from panel a)

Rhinovirus was the most commonly detected virus and was found in asymptomatic and symptomatic children (Fig. 3). Information regarding rhinovirus subtypes detected can be found in Additional file 4. The detection of any virus was associated with the presence of respiratory symptoms (*p* < 0.001). Rhinovirus was positively associated with the presence of respiratory symptoms (*p* < 0.001). Other viruses, such as RSV, HPIV and HMPV were detected mostly in symptomatic children (Table 2), and all were positively associated with the presence of respiratory symptoms (*p* < 0.001 for RSV and HPIV and HMPV *p* = 0.003) compared to those without any virus detected.

The four common circulating human coronaviruses (the alphacoronaviruses -229E and -NL63, and the betacoronaviruses -HKU1 and -OC43 (prior to the global spread of SARS-CoV-2)) were detected in oropharyngeal swabs and coronavirus-OC43 was detected in oral swabs at birth. Overall infection with any of the coronaviruses was significantly associated with the presence of respiratory symptoms (*p* = 0.011) when compared with those with no virus detected. Coronavirus infections throughout the first two years of life were relatively infrequent (up to 5.7% of swabs/timepoint (most frequent at 18 months)). The cohort was followed for a further year, and when extending analysis to 36 months visits, there were similarly low numbers of coronavirus infections of each subtype.

### Season and respiratory viral infection

There were more study visits during the Autumn months (Table 3). There were significantly more viral infections in Winter (*p* < 0.001) and Spring (*p* < 0.001) compared to the reference period of Summer. The percentage of infants who were symptomatic at the visit was higher in Winter (*p* < 0.001), Autumn (*p* = 0.001) and Spring (*p* = 0.006), compared to Summer. Rhinovirus was significantly more likely to be positive in Autumn (*p* = 0.007) and Spring (0.023) and Adenovirus was detected significantly less frequently in Autumn (*p* = 0.007). RSV peaked in Winter (*p* = 0.011), although overall, there were small numbers of RSV infections detected in this cohort. There was seasonality to the coronavirus infections with a significantly greater number of infections in Winter (*p* = 0.004) and Spring (*p* = 0.027).

**Table 3:** Virus detection on oropharyngeal swabs in each season for all samples to 2 years of age taken at routine visits (including those who did not complete follow-up excluding birth samples)

	**Winter**^**a**^	**Spring**	**Summer**	**Autumn**
Number of samples (%)	296 (25%)	305 (26%)	226 (19%)	367 (31%)
Symptomatic (%)	118 (40%)	102 (33%)	52 (23%)	131 (36%)
Mean age (days) at visit	312	364	335	263
Individual viruses total number positive (in brackets % symptomatic)				
Rhinovirus	135 (52%)	142 (47%)	80 (39%)	176 (48%)
Adenovirus	38 (61%)	49 (39%)	34 (44%)	26 (50%)
Bocavirus	25 (36%)	29 (52%)	9 (44%)	20 (55%)
Coronavirus	22 (41%)	14 (21%)	2 (50%)	4 (75%)
Polyomavirus	13 (46%)	35 (40%)	13 (23%)	11 (45%)
Metapneumovirus	7 (71%)	3 (67%)	2 (100%)	0 (0%)
RSV	19 (68%)	1 (100%)	1 (0%)	11 (82%)
Parainfluenza	4 (75%)	7 (71%)	2 (100%)	8 (75%)
Influenza	2 (50%)	2 (50%)	0	0

^a^Here the seasons are defined conventionally, as Winter (December, January, February), Spring (March, April, May), Summer (June, July, August), and Autumn (September, October, November)

### Multiple virus infections

The percentage of participants who had swabs simultaneously positive for multiple viruses increased with age (Additional file 5: S1 Fig). There were no swabs positive for multiple viruses at birth. In total, 201 swabs taken were positive for multiple viruses from routine visits. 57% of participants positive for multiple viruses had symptoms at the time of sampling, in comparison to 40% of participants whose swab was positive for a single virus. 176 (88%) of participants infected with multiple viruses were positive for rhinovirus.

### Associations between clinical factors and detection of respiratory virus

The association between respiratory virus infection and clinical factors was explored initially using univariate logistic regression analysis for those who continued in the study to 24 months (see Additional file 3: Table S5). Significant associations were found between age, respiratory symptoms, male sex, being in childcare, living with other children, season of sample and the dependent variable respiratory viral infection. This was further explored in a multivariable analysis using generalised estimating equations due to the longitudinal nature of the data. As illustrated in Table 4, male sex, season of sample, living with other children, attending childcare and respiratory symptoms were all associated with the detection of respiratory virus. When particularly exploring rhinovirus infection, male sex, living with other children, childcare and respiratory symptoms were associated with a positive swab for rhinovirus.

**Table 4:** The association between clinical factors and the presence of a respiratory virus on the oropharyngeal swab. The results from using Generalized Estimating Equations with an exchangeable correlation structure to explore associations of clinical factors and the presence of virus in the oropharyngeal swabs

**Variable**	**Coefficient**	**OR**	**CI**^b^**2.5%**	**CI**^b^**97.5%**	***P*value**
Male	0.51	1.67	1.23	2.27	**0.001**
Age	0.00	1.00	1.00	1.00	0.082
Complete months of breastfeeding	0.01	1.01	0.98	1.05	0.563
Season (reference Summer)^a^					
Autumn	0.34	1.40	0.96	2.04	0.077
Winter	0.58	1.78	1.22	2.60	**0.003**
Spring	0.56	1.75	1.20	2.55	**0.004**
Siblings/other children	0.46	1.59	1.15	2.19	**0.005**
Childcare	0.67	1.96	1.29	2.98	**0.002**
Respiratory symptoms	1.10	3.00	2.17	4.14	** < 0.001**

^a^Here the seasons are defined conventionally as, Winter (December, January, February), Spring (March, April, May), Summer (June, July, August), and Autumn (September, October, November)

^b^CI (confidence interval) Bold type indicates statistical significance with p < 0.05

When considering the detection of multiple viruses, age, respiratory symptoms and current childcare were all significantly associated with detection of multiple viruses when comparing to detection of single virus or negative swab.

### Early life wheeze and respiratory virus detection

There were 33 infants who were classified as having doctor-diagnosed wheeze by their 2 year review. For 20 infants there were insufficient data to classify with regard to wheeze, and 106 infants did not have doctor-diagnosed wheeze. The mean age of diagnosis was 356 days with a range 79 to 690 days and median of 311 days. Three of the 33 infants were diagnosed with wheeze under 6 months of age. One of these had a sample at 6 weeks visit (45 days) and no further samples until 6 months and had an episode of doctor-diagnosed wheeze at 79 days of age. One participant had an episode of doctor-diagnosed wheeze at 148 days, with preceding samples at 38 days (6 weeks visit), 99 days (3 months visit) and 134 days (4.5 months visit). One participant contributed a 6 weeks swab (50 days), and no 3 month or 4.5 months samples and had an episode of doctor-diagnosed wheeze at 148 days also.

A logistic regression model was used to explore any association between early respiratory viral infection (at our sample points) and doctor-diagnosed wheeze by 2 years of age. On univariate analysis there was no significant association between viral infection at 6 weeks, 3 months or 4.5 months and the outcome of doctor-diagnosed wheeze. When adjusting for clinical factors (sex, parental asthma, parental eczema, completed months of breastfeeding, living with other children, season of sampling and respiratory symptoms at time of sampling) there remained no significant association between detection of early respiratory virus and subsequent doctor-diagnosed wheeze. In addition, respiratory symptoms at each of the timepoints was not associated with this outcome.

### Exploration of breastfeeding and viral infection

There was a lack of association between completed months of breastfeeding and respiratory viral infection (Table 4). When assessing any association using the variable current feeding, there was no significant association at 6 weeks, and 3 months between feed type and viral detection by univariate analysis. At 4.5 months virus detection was associated with mixed feeding (*p* = 0.049).

When replacing completed months of breastfeeding with current feeding for all ages in the GEE in Table 4, current feeding was associated with presence of a virus – being weaned onto solids was positively associated with detection of virus (*p* = 0.002). For multiple viruses being weaned onto solids was also associated with greater detection of multiple viruses (*p* < 0.001).

Current feeding was not associated with whether there were symptoms or not during viral infection at 6 weeks, 3 months, or 4.5 months. Longitudinally, feeding at the time of the visit was not associated with whether there were symptoms when there was viral detection (GEE analysis all ages, adjusting for age, sex, season of sample, siblings/living with other children, childcare).

## Discussion

We found a temporal pattern of respiratory viral infection during the first months of life with increasing detection of respiratory viruses in the oropharynx to 9 months, following which there was a plateauing of infection. This is very similar to the temporal pattern detected previously using weekly nasal swabs [6]. There are several possible explanations for why the younger infants have a lower rate of viral infection. It may be due to the lower number of contacts that a younger baby may have compared to an older infant – of note childcare and living with other children were significantly associated with viral infection. Another explanation may be that it reflects transplacental transfer of antibodies which are protective against respiratory viral infection [14].

The detection of respiratory viruses was associated with respiratory symptoms, as has been shown previously [15]. Particular viruses such as rhinovirus showed a temporal trend and displayed both symptomatic and asymptomatic infection. Rhinovirus subtypes were determined in the earliest sample (at 6 weeks), and an expected pattern was found [16] with a similar frequency of swabs positive for subtypes A and C, and less swabs positive for subtype B (Additional file 4).

Some viruses such as HPIV and HMPV did not show a similar temporal trend and were mainly detected in symptomatic infants. Previous studies have found a similar correlation between age and presence of virus but not the variation with particular viruses [3, 17, 18]. It may be that the environmental niche of the epithelial lining of the oropharynx is more suited to rhinovirus and adenovirus infection, but disruption to this, for example with a change in the microbiota or another environmental or host factor, may predispose to infections with, for example, RSV, or to be symptomatic from rhinovirus infection itself [5, 19].

In the light of the current pandemic caused by SARS-CoV2, we consider the prevalence of coronaviruses in our cohort – a ‘pre-pandemic’ picture. Two alpha and two beta coronaviruses have been described as common causes of upper respiratory tract infection, and all four were detected in the oropharynx of asymptomatic children at a similar degree to that reported in previous studies in young children [8, 20] and with a similar seasonality to other studies [21]. Infections at birth were rare (1.4%). While our data suggest a relatively low level of infection in children to two years, more frequent sampling may have revealed a greater incidence of transient infection (noting serological studies have suggested up to 75% of young children have had a coronavirus infection by their 4^th^ birthday) [22, 23].

There is a general perception that breastfeeding is protective against respiratory tract infections. However, there is less robust evidence in the literature regarding a protective effect of breastfeeding on respiratory infection compared to gastrointestinal infection [24]. The relationship is more complex particularly in a high-income setting, potentially dependent on a longer period of breastfeeding and modulated by other factors such as the extent of exposure to viral infections (for example through childcare or living with siblings) [25], and the overall effect of breastfeeding is modest. In our cohort breastfeeding did not appear protective against either acquiring a respiratory viral infection or whether there were respiratory symptoms with this although we acknowledge we lacked power to refute an association, our study was not designed to look specifically at this association and our sampling moments were relatively infrequent. It may be that breastfeeding is protective against the development of more severe illness [26] from respiratory viral infection for example lower respiratory tract infection rather than upper respiratory tract infection or asymptomatic infection. In a similarly sized study to ours, Alexandrino et al. [27], found a relationship between breastfeeding duration and lower respiratory tract infection but not with upper respiratory infection. Wang et al., evaluated the association between breastfeeding and respiratory symptoms in a larger number of children in the community in the UK, and found limited evidence of a protective role of breastfeeding on respiratory infections in the first two years of life [28]. Whilst this study did not include swabbing to detect respiratory viruses, their overall conclusion supports our findings. In another study [29], assessing respiratory viral detection in patients admitted with respiratory viral infection, exclusive breastfeeding at the time of symptoms onset was positively associated with respiratory viral infection, whereas longer breastfeeding duration was protective. The authors speculated this was secondary to breastfed infants having increased contact time with their mother and were more at risk of mother to infant respiratory virus transmission.

We hypothesized that there would be a relationship between early viral infection and later wheeze on the basis that previous studies have shown an association between early bronchiolitis and asthma. However, there was no significant association between early (< 6 months) viral infection and doctor-diagnosed wheeze by 2 years of age. There was also no association found between early respiratory symptoms and this outcome. We acknowledge that we had limited sampling frequency which may have reduced our power to detect an association. Others have found an association between early bronchiolitis and subsequent development of wheeze or asthma (summarised in [18]), however asymptomatic and mildly symptomatic early respiratory viral infection may not have the same effect. The outcome definition we have used is also different – we have documented early life wheeze rather than asthma, in an unselected cohort. Jackson et al. [9], investigating the association between early viral induced wheezing and later asthma found the strongest relationship was between those who wheezed with rhinovirus infection in the third year of life and those who had asthma at 6 years of age. Future follow-up of our cohort will be useful to establish whether there is a link between early respiratory viral infection and asthma, however we expect that there needs to be a more significant clinical illness in the context of respiratory viral infection for there to be long term sequelae.

The study had several strengths. There was longitudinal follow-up with good retention, home visits ensured relatively complete data and the outcome variable of wheeze was obtained from medical notes rather than relying on parental report which is known to have its limitations [30]. There were some limitations to the study. The site of sampling (oropharyngeal as oppose to nasopharyngeal) may have affected detection of some respiratory viruses [31]. The report of symptoms is subjective, and reporting may vary between families. Potential interplay between different viruses has not been considered when relating incidence to clinical factors, but this study was not powered to detect these associations. The participants were born during different seasons and hence risk of infection would potentially have been different, although this has been taken into account in our analysis. As discussed, the follow-up is relatively short, and continued follow-up will allow us to detect associations with longer- term health outcomes i.e., asthma.

## Conclusions

In conclusion, respiratory virus detection increased over the first 9 months of life associated with respiratory virus symptoms, male sex and contact with other children. There was no association between early (up to 6 months) virus detection and the outcome doctor-diagnosed wheeze. Continuing follow-up is being undertaken to explore any association of early viral infection with later asthma.

## Supplementary Information

**Additional file 1: Table S1.** Multiplex panels. **Table S2.** Multiplex panel constituents, primer sources and reaction conditions used for respiratory virus PCR. **Table S3. **Primer design for detection of polyomaviruses. 

**Additional file 2: Table S4. **Data for exploring the association between early respiratory viral infection and later wheeze. 

**Additional file 3: Table S5.** Clinical variables and respiratory virus swab results for visits to 24 months. 

**Additional file 4.  **Rhinovirus subtypes.

**Additional file 5: Figure S1. **Percentage of participants who had a swab positive for either single or multiple viruses.

## Data Availability

All data used in the analyses presented in this article are included in the supplementary information files.

## Abbreviations

µl: Microlitres
C/S: Caesarean Section
DNA: Deoxyribonucleic acid
GEE: Generalised Estimating Equations
HMPV: Human metapneumovirus
HPIV: Human parainfluenza virus
PCR: Polymerase chain reaction
RNA: Ribonucleic acid
RSV: Respiratory syncytial virus
RT-PCR: Reverse transcriptase polymerase chain reaction
