# Supplementary material for: The temporal pattern and lifestyle associations of respiratory virus infection in a cohort study spanning the first two years of life
Source: BMC Pediatr. 2022 Mar 31;22:166. doi: 10.1186/s12887-022-03215-3 (PMC8967688; doi:10.1186/s12887-022-03215-3)
Supplement: Supplementary file 1 — Additional file 1: Table S1. Multiplex panels. Table S2. Multiplex panel constituents, primer sources and reaction conditions used for respiratory virus PCR. Table S3. Primer design for detection of polyomaviruses. [file 12887_2022_3215_MOESM1_ESM.docx]

Supporting information for Powell E, Sumner E, Shaw AG, Calvez R, Fink CG, Kroll JS. **The temporal pattern and lifestyle associations of respiratory virus infection in a cohort study spanning the first two years of life**

Regarding the PCR, Adenovirus, Bocavirus, polyomaviruses KI and WU were all run as uniplex assays.

For all other respiratory viruses, the first round reactions were performed as 2 multiplexes (Table S1):

- Multiplex 1 included the first round primers for FluA, FluB, hMPV, RSV and Paraflu 1-4
- Multiplex 2 included the first round primers for Rhinovirus, Enterovirus and all four coronaviruses (229E, OC43, NL63 and HKU1).

For each multiplex, the second round reactions were the split as follows:

- Multiplex1:
  - FluA and FluB
  - MPV and RSV
  - Paraflu 1, 2,3 and 4
- Multiplex 2:
  - Rhinovirus
  - Enterovirus
  - 229E, OC43, NL63 and HKU1

If any of the second round multiplex was positive, each target would then be tested separately to identify which specific virus was present.

**Table S1:** Multiplex panels

| 1^st^ Round | |  | 2^nd^ Round | |
| --- | --- | --- | --- | --- |
| Multiplex 1 | Flu A/B, MPV, RSV, ParaFlu1-4 |  | Multiplex 1.1 | Flu A/B |
|  |  |  | Multiplex 1.2 | MPV and RSV |
|  |  |  | Multiplex 1.3 | ParaFlu1-4 |
|  |  |  |  |  |
|  |  |  |  |  |
| Multiplex 2 | Rhino, Entero, Coronaviruses (229E, OC43, NL63 and HKU1) |  | Multiplex 2.1 | Rhino |
|  |  |  | Multiplex 2.2 | Entero |
|  |  |  | Multiplex 2.3 | 229E, OC43, NL63 and HKU1 |

**Table S2:** Multiplex panel constituents, primer sources and reaction conditions used for respiratory virus PCR.

| **Panel** | **Multiplex Target** | | **Oligonucleotide source (Ref.)** | **1st Round PCR** | **2nd Round PCR** |
| --- | --- | --- | --- | --- | --- |
|  |  |  |  |  |  |
| 1 | Influenza-A |  | Dingle et al. (2004)(1) |  |  |
|  | Influenza-B |  | Zhang and Evans (1991)(2) |  |  |
|  | hMPV |  | Maertzdorf et al. (2004)(3) |  |  |
|  | RSV |  | van Elden et al. (2003)(4) |  |  |
|  | Parainfluenza-1 | | Dingle et al. (2004)(1) |  |  |
|  | Parainfluenza-2 | | Echevarría et al. (1998)(5) |  |  |
|  | Parainfluenza-3 | | Echevarría et al. (1998)(5) |  |  |
|  | Parainfluenza 4 | | Aguilar et al. (2000)(6) | 45°C 10 min, 94°C 2 min | 95°C 100 s |
|  |  |  |  | then 30 cycles of | then 27 cycles of |
| 2 | Rhinovirus |  | Steininger et al. (2001)(7) | 95°C 20s, 60°C 20 s, 72°C 1 min | 95°C 20 s, 55°C 20 s, 74°C 20 s |
|  | Coronavirus-229E | | Bellau-Pujol et al. (2005)(8) |  |  |
|  | Coronavirus-OC43 | | Myint et al. (1994)(9) |  |  |
|  | Coronavirus-NL63 | | Gaunt et al. (2010)(10) |  |  |
|  | Coronavirus-HKU1 | | Gaunt et al. (2010)(10) |  |  |
|  |  |  |  |  |  |
| 3 | Enterovirus |  | Read et al. (1999)(11) |  |  |
|  |  |  |  |  |  |
| 4 | Bocavirus |  | Manning et al. (2006)(12) |  | 94°C 40 s |
|  |  |  |  | 94°C 40 s | then 30 cycles of |
|  |  |  |  | then 30 cycles of | 95°C 20 s, 50°C 20 s, 72°C 20 s |
| 5 | Adenovirus |  | Allard et al. (2001)(13) | 95°C 20 s, 50°C 20 s, 72°C 20 s | 94°C 40 s |
|  |  |  |  |  | then 30 cycles of |
|  |  |  |  |  | 95°C 20 s, 55°C 20 s, 72°C 20 s, 82 °C 10 s |
| 6 | KI polyomavirus | | This study: |  |  |
|  |  |  | CTGTTGATGCACAGGTTGGT (outer sense) |  |  |
|  |  |  | GCTATGTTGTTATTGCTAATATGGT (outer antisense) |  |  |
|  |  |  | TTCAGTCCCAGRCTCCTTTG (inner sense) |  |  |
|  |  |  | CCTGTAAGTGACTTTGATGAAGAAA (inner antisense) |  |  |
|  |  |  |  | 94°C 40 s | 95°C 120 s |
| 7 | WU polyomavirus | | This study: | then 25 cycles of | then 30 cycles of |
|  |  |  | GCTAGGAMTTGCTCCCACTG (outer sense) | 95°C 20 s, 55°C 20 s, 72°C 20 s | 96 °C 0 s, 60 °C 10 s, 72.5 °C 20 s |
|  |  |  | GGGCCCTGTTTCTTCAGTCA (outer antisense) |  |  |
|  |  |  | AAGTACAGYATYGAATCATGGG (inner sense) |  |  |
|  |  |  | CCAACCATTCTGCCAAAGTAT (inner antisense) |  |  |
|  |  |  |  |  |  |

**Table S3:** Primer design for detection of polyomaviruses.

| Polyomavirus | Primer design |
| --- | --- |
| KI polyomavirus | The primers for KI polyomavirus detection were designed in-house using the NCBI Primer Design tool (<http://www.ncbi.nlm.nih.gov/tools/primer-blast/>) and the complete genome sequence from the KI polyomavirus Stockholm 60 (gi\|124366173\|gb\|EF127906.1\|). The target is a region located between the VP1 and the small T antigen of the virus. Primer sequences (5’>3’) were CTGTTGATGCACAGGTTGGT (F1) and GCTATGTTGTTATTGCTAATATGGT (R1) for the first round and TTCAGTCCCAGRCTCCTTTG (F2) and CCTGTAAGTGACTTTGATGAAGAAA (R2) for the second round. |
| WU polyomavirus | The primers for WU polyomavirus detection were designed in-house using the NCBI Primer Design tool (<http://www.ncbi.nlm.nih.gov/tools/primer-blast/>) and the complete genome sequence from the WU Polyomavirus strain B0 complete genome sequence (gi\|146199082\|gb\|EF444549.1\|).The target is a region located within the VP1 gene of the virus. Primer sequences (5’>3’) were GCTAGGAMTTGCTCCCACTG (F1) and GGGCCCTGTTTCTTCAGTCA (R1) for the first round and AAGTACAGYATYGAATCATGGG (F2) and CCAACCATTCTGCCAAAGTAT (R2) for the second round. |

**References**

1. Dingle KE, Crook D, Jeffery K. Stable and noncompetitive RNA internal control for routine clinical diagnostic reverse transcription-PCR. J Clin Microbiol. 2004;42(3):1003-11.

2. Zhang WD, Evans DH. Detection and identification of human influenza viruses by the polymerase chain reaction. J Virol Methods. 1991;33(1-2):165-89.

3. Maertzdorf J, Wang CK, Brown JB, Quinto JD, Chu M, de Graaf M, et al. Real-time reverse transcriptase PCR assay for detection of human metapneumoviruses from all known genetic lineages. J Clin Microbiol. 2004;42(3):981-6.

4. van Elden LJ, van Loon AM, van der Beek A, Hendriksen KA, Hoepelman AI, van Kraaij MG, et al. Applicability of a real-time quantitative PCR assay for diagnosis of respiratory syncytial virus infection in immunocompromised adults. J Clin Microbiol. 2003;41(9):4378-81.

5. Echevarria JE, Erdman DD, Swierkosz EM, Holloway BP, Anderson LJ. Simultaneous detection and identification of human parainfluenza viruses 1, 2, and 3 from clinical samples by multiplex PCR. J Clin Microbiol. 1998;36(5):1388-91.

6. Aguilar JC, Perez-Brena MP, Garcia ML, Cruz N, Erdman DD, Echevarria JE. Detection and identification of human parainfluenza viruses 1, 2, 3, and 4 in clinical samples of pediatric patients by multiplex reverse transcription-PCR. J Clin Microbiol. 2000;38(3):1191-5.

7. Steininger C, Aberle SW, Popow-Kraupp T. Early detection of acute rhinovirus infections by a rapid reverse transcription-PCR assay. J Clin Microbiol. 2001;39(1):129-33.

8. Bellau-Pujol S, Vabret A, Legrand L, Dina J, Gouarin S, Petitjean-Lecherbonnier J, et al. Development of three multiplex RT-PCR assays for the detection of 12 respiratory RNA viruses. J Virol Methods. 2005;126(1-2):53-63.

9. Myint S, Johnston S, Sanderson G, Simpson H. Evaluation of nested polymerase chain methods for the detection of human coronaviruses 229E and OC43. Mol Cell Probes. 1994;8(5):357-64.

10. Gaunt ER, Hardie A, Claas EC, Simmonds P, Templeton KE. Epidemiology and clinical presentations of the four human coronaviruses 229E, HKU1, NL63, and OC43 detected over 3 years using a novel multiplex real-time PCR method. J Clin Microbiol. 2010;48(8):2940-7.

11. Read SJ, Kurtz JB. Laboratory diagnosis of common viral infections of the central nervous system by using a single multiplex PCR screening assay. J Clin Microbiol. 1999;37(5):1352-5.

12. Manning A, Russell V, Eastick K, Leadbetter GH, Hallam N, Templeton K, et al. Epidemiological profile and clinical associations of human bocavirus and other human parvoviruses. J Infect Dis. 2006;194(9):1283-90.

13. Allard A, Albinsson B, Wadell G. Rapid typing of human adenoviruses by a general PCR combined with restriction endonuclease analysis. J Clin Microbiol. 2001;39(2):498-505.
