# Supplementary material for: The temporal pattern and lifestyle associations of respiratory virus infection in a cohort study spanning the first two years of life
Source: BMC Pediatr. 2022 Mar 31;22:166. doi: 10.1186/s12887-022-03215-3 (PMC8967688; doi:10.1186/s12887-022-03215-3)
Supplement: Supplementary file 4 — Additional file 4. Rhinovirus subtypes. [file 12887_2022_3215_MOESM4_ESM.docx]

Additional file 4: Rhinovirus subtypes

## Rhinovirus subtypes

For the 6 week samples, rhinovirus positive results were examined in more detail with subtyping for rhinovirus. Of the 204 oropharyngeal samples taken at 6 weeks of age, 59 samples were positive for rhinovirus. Samples were taken forward (at random) for sequencing in 46 cases. Sequencing was conducted in 46 yielding usable data in 34 cases. Rhinovirus A was detected in 16 swabs (47% of those in which sequencing was achieved), Rhinovirus B was detected in 4 swabs (12%) and Rhinovirus C in 13 (38%), additionally one swab was positive for both rhinovirus A and C.
