# Supplementary figures and images for: The temporal pattern and lifestyle associations of respiratory virus infection in a cohort study spanning the first two years of life
Source: BMC Pediatr. 2022 Mar 31;22:166. doi: 10.1186/s12887-022-03215-3 (PMC8967688; doi:10.1186/s12887-022-03215-3)

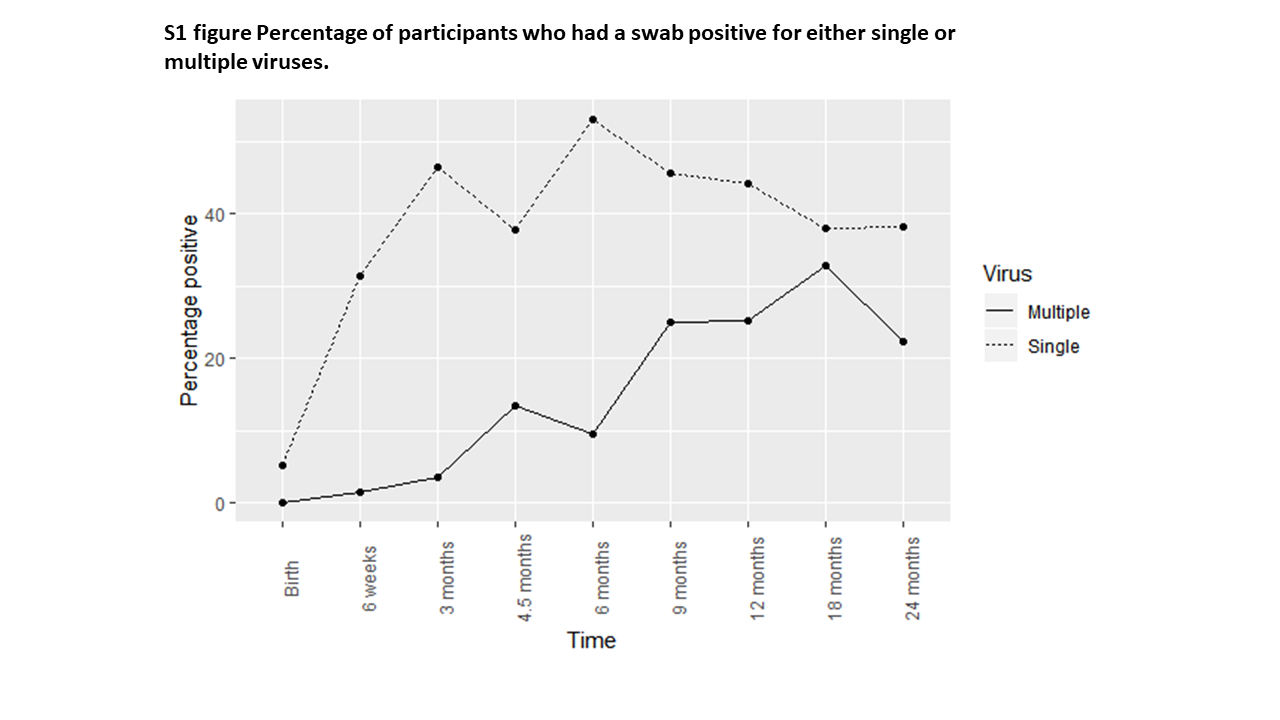

Supplement: Supplementary file 5 — Additional file 5: Figure S1. Percentage of participants who had a swab positive for either single or multiple viruses. [file 12887_2022_3215_MOESM5_ESM.tif]
